# First Case of Staphylococci Carrying Linezolid Resistance Genes from Laryngological Infections in Poland

**DOI:** 10.3390/pathogens10030335

**Published:** 2021-03-13

**Authors:** Michał Michalik, Maja Kosecka-Strojek, Mariola Wolska, Alfred Samet, Adrianna Podbielska-Kubera, Jacek Międzobrodzki

**Affiliations:** 1 MML Medical Centre, Bagno 2, 00-112 Warsaw, Poland; m.michalik@mml.com.pl (M.M.); dr.alfredsamet@gmail.com (A.S.); adrianna.podbielska@mml.com.pl (A.P.-K.); 2 Department of Microbiology, Faculty of Biochemistry, Biophysics and Biotechnology, Jagiellonian University, Gronostajowa 7, 30-387 Kraków, Poland; mariola.wolska@doctoral.uj.edu.pl (M.W.); jacek.miedzobrodzki@uj.edu.pl (J.M.)

**Keywords:** antibiotic resistance, *Staphylococcus aureus*, *Staphylococcus haemolyticus*, chronic sinusitis, laryngological infections

## Abstract

Linezolid is currently used to treat infections caused by multidrug-resistant Gram-positive cocci. Both linezolid-resistant *S. aureus* (LRSA) and coagulase-negative staphylococci (CoNS) strains have been collected worldwide. Two isolates carrying linezolid resistance genes were recovered from laryngological patients and characterized by determining their antimicrobial resistance patterns and using molecular methods such as *spa* typing, MLST, SCC*mec* typing, detection of virulence genes and *ica* operon expression, and analysis of antimicrobial resistance determinants. Both isolates were multidrug resistant, including resistance to methicillin. The *S. aureus* strain was identified as ST-398/t4474/SCC*mec* IVe, harboring adhesin, hemolysin genes, and the *ica* operon. The *S. haemolyticus* strain was identified as ST-42/*mecA*-positive and harbored hemolysin genes. Linezolid resistance in *S. aureus* strain was associated with the mutations in the ribosomal proteins L3 and L4, and in *S. haemolyticus*, resistance was associated with the presence of *cfr* gene. Moreover, *S. aureus* strain harbored *optrA* and *poxtA* genes. We identified the first case of staphylococci carrying linezolid resistance genes from patients with chronic sinusitis in Poland. Since both *S. aureus* and CoNS are the most common etiological factors in laryngological infections, monitoring of such infections combined with surveillance and infection prevention programs is important to decrease the number of linezolid-resistant staphylococcal strains.

## 1. Introduction

Linezolid, the first oxazolidinone antimicrobial approved in clinical practice, is currently used to treat infections caused by Gram-positive cocci, especially methicillin-resistant *Staphylococcus aureus* (MRSA) and vancomycin-resistant enterococci [1]. The first linezolid-resistant *S. aureus* (LRSA) strain was detected in 2001 [2]. Since then, linezolid-resistant *S. aureus* strains have been collected worldwide, but most of them are related to (i) severe diseases such as cystic fibrosis [3,4], (ii) ICU patients [5,6] or (iii) chronic infections with long-term linezolid treatments [7]. Not only have *S. aureus* linezolid-resistant strains been reported, but increasing linezolid resistance in coagulase-negative staphylococci (CoNS) strains has also been observed. The first linezolid-resistant *S. haemolyticus* (LRSH) strain was reported by Rodríguez-Aranda et al. in 2009 [8]. Since then, a few strains (namely, 11) have been reported worldwide [9,10], but most of them were reported from China [11,12] and from India [13,14,15], with the last one in 2019 [16].

Linezolid reversibly binds and blocks the ribosomal peptidyl transferase center (PTC) and by this mechanism exerts bacteriostatic activity [17]. In staphylococcal clinical isolates, the mutation of the V domain of the 23S rRNA, namely, G2576U, is the most common modification of the ribosome at the PTC [2,9], but other mutations have also been identified [18]. Linezolid resistance has also been associated with mutations in the L3, L4, and L22 ribosomal proteins [18,19,20]. Moreover, the linezolid resistance, a transferable one, may be related to the *cfr* gene known since year 2000, and in staphylococci, firstly discovered in a bovine *Staphylococcus sciuri* strain and later reported also in other staphylococcal species [21]. The *cfr* gene is not only responsible for resistance to oxazolidinones but also mediates cross-resistance to other antibiotics, such as phenicols, lincosamides, pleuromutilins, and streptogramin A [22]. Recently, the linezolid resistance was also associated with the novel transferable oxazolidinone resistance gene, namely *optrA*. First, it was identified, mainly in enterococci from humans and animals [23,24] but recently, the *optrA* gene was detected also in a single porcine *S. sciuri* strain [25,26] and later confirmed in a few other *S. sciuri* strains [27]. In contrast to *cfr* gene, *optrA* confers cross-resistance only to oxazolidinones, including tedizolid and phenicols. In 2018, Antonelli et al. described the novel gene, named *poxtA,* responsible also for transferable linezolid resistance in MRSA strains. The *poxtA* gene encodes a protein of the ARE ABC-F family (lineage F of the ABC superfamily proteins associated with antibiotic resistance), one of the ribosomal protection proteins [28]. The *poxtA* gene is distantly related to *optrA* and able to cross-mediate susceptibility to phenicols, oxazolidinones, and tetracyclines. Moreover, it was also observed that *poxtA* gene could act synergistically with other oxazolidinone resistance mechanisms to further increase the level of resistance to this group of antibiotics [28].

Linezolid resistance has also emerged in patients without linezolid exposure, which is probably due to cross-transmission between patients, horizontal transfer of linezolid resistance mediated by transferable genes among different CoNS species or co-selection by treatment with other antibiotics [15,22]. Moreover, linezolid-resistant strains are also resistant to other groups of antibiotics, especially linezolid resistant strains are often simultaneously resistant to β lactams, so a proper characterization of broad resistance mechanisms is required [29].

In Poland, the only study related to linezolid resistant *Staphylococcus* strains recovered from ICU patients was published by our group in 2020 [6]. To date, there are no published reports related to linezolid-resistant strains detected in laryngological infections. Therefore, the study presented is the first in that field. Considering that staphylococci, including both *S. aureus* and CoNS, are the most frequent etiological factors in laryngological infections [30], their genetic and antimicrobial resistance profiles need to be further evaluated.

In the present study, two *Staphylococcus* isolates from laryngological infections were evaluated for their mechanisms of linezolid resistance and genetic profiles, and linked to patient characteristics.

## 2. Results

### 2.1. Patient Characteristics

Two patients hospitalized in the MML Center were evaluated. The first patient, a male of age 37, was diagnosed with chronic sinusitis. In 2013, the patient underwent nasal septum correction, correction of lower nasal turbinates by the Celon method, functional endoscopic surgery of the paranasal sinuses, removal of a foreign body from the left maxillary sinus, and correction of the soft palate by the Celon method. Then, in 2016, the patient was admitted to a clinic with purulent runny nose after dental treatment and diagnosed with chronic maxillary sinusitis. The patient was referred for functional endoscopic sinus surgery (FESS). The *S. haemolyticus* (WAW954 isolate) was cultured from the right sinus.

The second patient, a male aged 57, was admitted to the MML Center in the middle of 2016, diagnosed with chronic sinusitis, and qualified for surgery. The *S. aureus* (WAW1257 isolate) was cultured from right and left maxillary sinuses. During laryngological procedures, none of the patients were treated with linezolid. However, the first patient was treated with amoxycillin, clavic acid, and co-trimoxazole and the second patient with rifampicin and fusidic acid.

### 2.2. Characteristics of Isolates and Identification at the Species Level

A set of two isolates WAW1257 and WAW954 from the maxillary sinus collected from patients treated in the MML Medical Center were investigated. The preliminary identification with the Vitek^®^ 2 system identified the WAW1257 isolate as *S. aureus* and WAW954 as *S. haemolyticus*. Species identification was confirmed by four Sanger sequencing methods, namely, 16S rRNA, *sodA, tuf,* and *rpoB* genes.

### 2.3. Genetic Profiling and Clonality Analysis

Multilocus sequence typing (MLST) analysis revealed that *S. aureus* WAW1257 was ST398 and *S. haemolyticus* WAW954 was ST42. The *S. aureus* strain was assigned as *spa* type t4474. The *SCCmec* typing methods allowed for the identification of the *SCCmec* type IV subtype E for the WAW1257 strain and the presence of only the *mecA* gene in WAW954. The arginine catabolic mobile element (ACME) typing showed that WAW1257 contained ACME type II (*arc*+, *opp3*−) and WAW954 ACME type III (*arc*−, *opp3*+) (Table 1).

### 2.4. Virulence and Biofilm Formation Genes

The *S. aureus* (WAW1257) strain was positive for the *clfB*, *clfA*, *fnbB*, *fib*, *hlg*, *hla*, *hld*, and *hlb* genes, and *S. haemolyticus* WAW954 was positive for the *fib*, *hla,* and *hlb* genes. Additionally, the *S. aureus* strain harbored the *icaABDC* operon (Table 1).

### 2.5. Antimicrobial Susceptibility and Resistance Determinants

Based on the European Committee on antimicrobial susceptibility testing (EUCAST) breakpoints tables, the *S. haemolyticus* isolate was susceptible to only four antibiotics, tested in this study. Both isolates exhibited susceptibility to daptomycin and amikacin. Moreover, the *S. aureus* isolate was susceptible to fosfomycin, tigecycline, gentamicin, and *S. haemolyticus* to vancomycin and trimethoprim-sulfamethoxazole (Table 2). The linezolid resistance was tested with the E-test method and resulted in MIC = 3 µg/mL for *S. aureus* and MIC = 6 µg/mL for *S. haemolyticus* (Table 2). Therefore, *S. aureus* was assigned as linezolid susceptible and *S. haemolyticus* as resistant.

In our study, both *S. aureus* and *S. haemolyticus* strains exhibited intermediate levels of resistance to vancomycin, MIC = 3 µg/mL for *S. aureus* and MIC = 4 µg/mL for *S. haemolyticus*. Therefore, both isolates were reported as vancomycin intermediate *S. aureus* (VISA) or vancomycin intermediate *Staphylococcus* sp. (VISS). Both *S. aureus* and *S. haemolyticus* strains were resistant to teicoplanin (Table 2). The *S. haemolyticus* isolate was also resistant to chloramphenicol and clindamycin, consistent with the presence of the *cfr* and *fexA* genes. The occurrence of *cfr* gene mediates in rendering the so-called PhLOPSA phenotype. *S. haemolyticus* was resistant to gentamicin, as confirmed by the presence of the *aac(6′)-Ie-aph(2″)* gene. The *S. aureus* strain was resistant to ciprofloxacin and levofloxacin, and had the *norA* gene (Table 2). Additionally, the *S. aureus* strain demonstrated T314C and G362A changes in their deduced amino acid sequences of the L3 protein and C575T changes in their deduced amino acid sequences of the L4 protein. For *S. aureus* and *S. haemolyticus* strains, no changes occurred in the analyzed part of the 23S rRNA genes or in the L22 protein genes. For the *S. haemolyticus* strain, no changes occurred in the L3 or L4 protein genes. Moreover, the *S. aureus* strain harbored the *optrA* and *poxtA* genes (Table 2). Altogether, the strains were resistant to six various classes of antimicrobials, i.e., they were multidrug resistant.

## 3. Discussion

In the era of multidrug-resistant strains, linezolid is still an effective treatment agent for Gram-positive coccus infections [31]. Nevertheless, the increase in linezolid-resistant *S. aureus*, *S. haemolyticus,* and other CoNS is worrisome. Mostly, the number of linezolid-resistant strains occurs after increased administration of an antibiotic but not always [3,4,29]. What is important, the linezolid resistance can emerge in CoNS after only a few days of treatment and in *S. aureus* strains, it usually occurs after a long time after the treatment [18].

In our study, to our knowledge, the patients were not exposed to linezolid prior to the isolation of the resistant strains. This acquisition of linezolid resistance may relate to the highly plastic nature of the CoNS genome, which is driven largely by insertion sequences and other mobile genetic elements [32]. Patients may have acquired the strains carrying the linezolid resistance genes from their environment, during their other hospital stay or could have also undergone the linezolid treatment due to infections other than sinusitis. Here, we describe the first cases of *S. aureus* and *S. haemolyticus* strains carrying linezolid resistance genes collected from patients with chronic sinusitis. In recent years, sinus infections have developed into chronic maxillary sinusitis over time in approximately 15% of patients [33]. The occurrence of multidrug-resistant strains in such patients is a next step in spreading antibiotic resistance, including the one for so-called last chance antibiotics such as vancomycin. In case of our strains, the divergent results of vancomycin resistance testing were most probably due to hetero-resistance [34]. Based on previous research, the precise cut-off values for both VISA and VISS phenotypes change with time and are different depending on the country [35]. Due to KORDL recommendations [36], we believe that our MIC values, which are slightly over breakpoint, can be considered as vancomycin-intermediate.

Unlike prior studies, where ST5 and ST188 were predominant among linezolid-resistant strains (data for *S. aureus*) [3,37], in our study, the *S. aureus* strain belonged to ST398, one of the most frequent lineages of LA-MRSA in Europe [38,39]. It was observed that the occurrence of LA-MRSA in human is strongly associated with the increased contact with livestock [40]. Furthermore, it is known that ST398 often shows extensive resistance, which is selected by the widespread use of antibiotics in livestock farming [41,42]. In the present case, the patient affirmed that he was engaged in animal breeding activities (cattle and poultry) and lived in proximity to dogs and cats.

As reported previously, ST398 can be combined with SCC*mec* type IV [43,44], and such a situation also occurred in our study. Due to previous studies, it was reported that the *SCCmec* IV has smaller components and due to its increased mobility was found in different genetic backgrounds [45]. Moreover, SCC*mec* IV is mostly related to community-acquired MRSA (CA-MRSA) strains and is rarely found in health-care-associated MRSA strains (HA-MRSA) [46]. In recent years, it was also observed that SCC*mec* IV is present in several HA-MRSA clones, especially in Europe [47,48] but also worldwide [49,50]. Recent studies have reported that CA-MRSA strains are spreading in hospital settings and are replacing traditional HA-MRSA strains, especially in the United States of America [51,52].

To date, only a few reports on t4474 have been published. Data from one *S. aureus* strain belonging to t4474 isolated in Switzerland were submitted to a Ridom Spa Server database. Ho et al. published a study concerning MRSA from slaughtered pigs sampled from local markets in Hong Kong in 2012 [53], and Rodríguez-López et al. characterized MRSA from the Italian heavy swine production chain in 2020 [54]. Therefore, the worldwide distribution of this particular *spa* type is not exactly known. In our study, the *S. aureus* strain belonged to t4474, which is also consistent with the fact that the patient had contact with animals.

The arginine catabolic mobile element (ACME) was first described in methicillin-resistant *Staphylococcus aureus* and is considered to enhance transmission, persistence, and survival. It was shown that ACME elements are especially prevalent in CoNS species [55]. Considering that not only the ACME is associated with the widespread *S. aureus* clones but also its high prevalence in *S. epidermidis* strains was noted [56], we believe that the detection of this element in *S. aureus* and CoNS strains from laryngological infections, where the strains must survive sometimes for a long time, is crucial for monitoring the transmission and better understanding such strains. ACME is integrated downstream of the SCC*mec* cassette and is flanked by repeat sequences, together with cassette chromosome recombinase (*ccr*) genes. It was proven, that *ccr* genes catalyze the integration and excision of ACME from the staphylococcal chromosome [56], which is consistent with our study, that the ACME element coexists with *SCCmec* type IV [57,58,59]. In contrast to studies performed on *S. aureus*, the ACME in CoNS has not yet been thoroughly clarified [60]. Previously, ACME types were distinguished by characteristic presence profiles of the *arc* and *opp3* operons but recently, two novel ACME types harboring the potassium transporter-encoding operon *kdp* were described. In our study, the *S. aureus* strain possessed ACME type II, and the *S. haemolyticus* strain had ACME type III.

In our study, all the strains harbored virulence factors related to adhesion and hemolysis processes. In laryngological infections, not only are the adhesion-related factors important but also enzymes such as hemolysins can play a role in some of the effects of staphylococci on host organisms, with both involved in tissue destruction and as spreading factors facilitating invasion into nearby tissues [30]. Moreover, these factors may also be related to strains persistence in a host. In our study, the *S. aureus* strain also carried the *icaADBC* locus, which is responsible for the production of polysaccharide intercellular adhesin (PIA), playing an important role in biofilm formation by bacteria [61]. In ICU patients with MRSA respiratory infection intubated for long periods, the systemic treatment with linezolid has a beneficial effect in limiting the MRSA burden [62,63]. Independent of that phenomenon, generally, the circulation of linezolid-resistant strains within a biofilm-associated operon generates a great risk for patients.

Although *cfr*-positive MRSA strains have occurred in many *S. aureus* lineages, in our study, the MRSA strain was *cfr*-negative and did not harbor mutations in the V region of 23S rRNA. In our study, the *S. aureus* strain had two mutations in the L3 protein (T314C; G362A) and one in the L4 protein (C575T). The amino acid substitutions were detected based on a comparison with reference genomes of linezolid-sensitive *S. aureus* strains. The obtained sequences were compared to the reference *rplC* and *rplD* gene sequences of *S. aureus* NCTC8325, N315, and MW2. We believe that the unambiguous confirmation of these mutations should be determined by whole genome sequencing, which can be performed in the future. Moreover, the *S. aureus* strain harbored both *optrA* and *poxtA* genes, responsible for transferable linezolid resistance. To our knowledge, this is the first detection of both *optrA* and *poxtA* genes and L3/L4 mutations in a single strain.

*S. haemolyticus* is a part of natural human skin microbiota and is, after *S. epidermidis*, the second most frequent species among clinical isolates of CoNS [64]. Nowadays, this species is recognized as an important nosocomial pathogen with a drift to develop multiple drug resistance, probably due to insertion sequences in its chromosome resulting in genomic rearrangements [65]. Indeed, *S. haemolyticus* was the first one among Gram-positive pathogens which acquired glycopeptide resistance and seems to show increased teicoplanin resistance in comparison to other CoNS [66]. In our study, the *S. haemolyticus* strain had only the *mecA* gene. Although, such structure of the SCC*mec* cassette was confirmed by two independent SCC*mec* typing methods, this is either a situation that the corresponding SCC*mec* element was non-typeable due to the alternative structure or modified primer binding sites or only the *mecA* gene is present. However, Miragaia et al. 2018 described that the CoNS species, including *S. haemolyticus* were characterized by high genetic diversity and recombination rate. Moreover, the ability to acquire and maintain exogenous genetic material or genetic mobile elements have been acquired earlier by these species than by *S. aureus* strains [67]. What is worrisome, the infection prevention controls, which are administered for MRSA are not used for CoNS and as a result, many multidrug resistant isolates, even those resistant to linezolid, stay undetected in health care settings. The detection of linezolid resistance in *S. haemolyticus* strains seems to be an emerging issue and requires stricter control to preserve linezolid for its clinical utility.

In our study, *S. haemolyticus* was PCR-positive for the *cfr* and *fexA* genes. The *fexA* gene presence was consistent with chloramphenicol resistance and the *cfr* gene detection conferred the *S. haemolyticus* PhLOPS_a_ phenotype [22]. The *cfr* gene is located either in the chromosome or in plasmids or transposons which indicates a higher ability to transfer between strains [68,69]. The spread to susceptible populations or other pathogenic bacteria is facilitated. Moreover, the *cfr*-mediated resistance is related to an array of other antibiotics which limits therapeutic options. In *Staphylococcus*, the *fexA* gene is located in a small transposon Tn558 or in combination with the *cfr* gene in transposition-deficient Tn558 variants [70]. Here, we link the *S. haemolyticus* resistance with the presence of *cfr* gene, as no mutation in 23S rRNA nor L3/L4/L22 proteins was found. Such situation was observed for other linezolid resistant CoNS species [4,15,22].

To conclude, we identified the first cases of multidrug resistant *S. aureus* strain carrying linezolid resistance genes and linezolid-resistant *S. haemolyticus* strain from patients with chronic sinusitis in Poland. Since *S. aureus* and CoNS are the most common etiological factors of laryngological infections, monitoring linezolid resistance, together with the genetic characterization of such strains, is an emerging issue.

## 4. Materials and Methods

### 4.1. Strain Collection

The set of bacterial isolates used in this study included *S. aureus* (WAW1257) and *S. haemolyticus* (WAW954) clinical isolates carrying linezolid resistance genes recovered in 2016 from laryngological patients treated in MML Medical Center, Warsaw. Both isolates were recovered from maxillary sinuses. The preliminary identification of isolates was performed with a Vitek^®^ 2 Compact instrument (bioMérieux, La Balme Les Grottes, France).

### 4.2. Susceptibility Testing

Susceptibility testing was carried out according to the European Committee on Antimicrobial Susceptibility Testing (EUCAST; www.eucast.org/; accessed on 26 February 2021) recommendations. Minimum inhibitory concentration (MIC) values for linezolid, cefoxitin, vancomycin, teicoplanin, daptomycin, fosfomycin, ciprofloxacin, tetracycline, tigecycline, chloramphenicol, gentamicin, clindamycin, amikacin, erythromycin, trimethoprim-sulfamethoxazole, levofloxacin, nitrofurantoin, and benzylpenicillin were determined using the E-test method.

### 4.3. Total DNA Extraction

For genomic DNA extraction, isolates were grown for 20 h at 37 °C on blood agar plates. A full inoculation loop of 10 μL of bacterial colonies was homogenized with a TissueLyser II (Qiagen, Germantown, MD, USA). The Qiagen DNeasy Blood and Tissue Kit (Qiagen, Germantown, MD, USA) was used for genomic DNA extraction. The subsequent steps were performed according to the manufacturer’s instructions. Purified DNA was stored at −20 °C.

### 4.4. Species Identification

Both isolates were identified at the species level by sequencing the 16S rRNA, *sodA*, *tuf,* and *rpoB* genes, as previously described [71,72,73,74]. The PCR products were resolved by electrophoresis and purified using the Clean-Up Concentrator purification kit (A&A Biotechnology, Gdynia, Poland). The concentration and purity were measured using a NanoDrop ND-1000. The PCR products were sequenced with the Sanger method at Genomed S.A. (Warsaw, Poland) with the same primers as those used for PCR.

### 4.5. Molecular Analysis

#### 4.5.1. *Spa* Typing

*Spa* typing, based on the amplification of the variable X region of the protein A gene, was performed as described previously [75]. After sequencing, the *spa* type was assigned using the Ridom StaphType software version 2. 2. 1 (Ridom GmbH, Würzburg, Germany) and the Ridom SpaServer (https://spaserver.ridom.de/; accessed on 25 May 2020).

#### 4.5.2. MLST Typing

The clonality of isolates was studied using multilocus sequence typing (MLST) [76,77]. All PCR products were sequenced, and the *S. aureus* and *S. haemolyticus* MLST websites (https://pubmlst.org/shaemolyticus/; accessed on 25 May 2021, https://pubmlst.org/saureus/; accessed on 25 May 2020) were used to assign alleles and sequence types (STs) for allelic profiles [78].

#### 4.5.3. *SCCmec* Cassette Typing

The *SCCmec* cassettes were typed with two independent methods, as described previously by Milheirico et al. [79] and Kondo et al. [80], with the USA300 strain as a positive control for the IV *SCCmec* cassette. The PCR products were resolved by electrophoresis, and the band patterns were analyzed.

#### 4.5.4. ACME Cassette Typing

The presence of ACME cassettes in the *S. aureus* strain was detected by multiplex PCR targeting the *arcA* (AIPS27, AIPS28) and *opp3A* (AIPS45, AIPS46) genes using a previously described protocol [81] (*arcA* AIPS27 5′-CTAACACTGAACCCCAATG-3′; AIPS28 5′-GAGCCAGAAGTACGCGAG-3′), (*opp3A* AIPS45-5′-GCAAATCTTAAATGGTCTGTTC-3′; AIPS46 5′-GAAGATTGGCAGCACAAAGTG-3′). Single PCR targeting the *arcA* and *opp3B* (opp3B-F, opp3B-R) genes was performed as previously described by O’Connor et al. [82] for *S. haemolyticus* (*opp3B* opp3B-F 5′-GGATTCGCCCAAGTGATGACC-3′; opp3B-R 5′-GACTGCTGGGTATGACGT-3′). The PCR products were resolved by electrophoresis, and the band patterns were analyzed.

#### 4.5.5. Detection of Virulence and *ica* Operon Genes

The PCRs for the detection of virulence determinants such as adhesins, hemolysins, and biofilm formation genes were performed as described in Table 3. The PCR products were resolved by electrophoresis, and the band patterns were analyzed.

#### 4.5.6. Detection and Analysis of Antimicrobial Resistance Determinants

Detection of the *cfr, fexA, norA, aac(6)-Ie-aph(2″)* genes was performed as previously described [5,6,70,83,84,85,86]. Additionally, the presence of *optrA* and *poxtA* genes was checked [23,87]. All the PCR products were resolved by electrophoresis, and the band patterns were analyzed.

The genes encoding the PTC-associated ribosomal proteins L3 (*rplC*), L4 (*rplD*), L22 (*rplV*), and 23S rRNA were amplified with the primers and PCR conditions described in Table 4. The PCR products were cleaned and concentrated with a Clean-Up Concentrator purification kit (A&A Biotechnology, Gdynia, Poland) and sequenced (Genomed S.A., Warsaw, Poland) with primers for individual ribosomal protein genes. The obtained sequences were compared to the reference *rplC*, *rplD, rplV,* and 23S rRNA gene sequences for *S. haemolyticus* JCSC1435 (GenBank accession number: NC_007168.1) and *S. aureus* NCTC8325, N315 and MW2 (GenBank accession numbers: NZ_LS483365.1, NC_002745.2, and NC_003923.1).

### 4.6. Nucleotide Sequence Accession Numbers

The eight sequences for one *Staphylococcus haemolyticus* and one *Staphylococcus aureus* were annotated using the NCBI BankIt tool and deposited in the GenBank database (https://www.ncbi.nlm.nih.gov/genbank/) under the following accession numbers: For the 16S rRNA gene, MW267294 and MW267295; for the *sodA* gene, MW272559 and MW272560; for the *tuf* gene, MW272562 and MW272563; and for the *rpoB* gene, MW272556 and MW272557.

## Figures and Tables

**Table 1:** Genetic profiles of *S. aureus* and *S. haemolyticus* strains isolated from laryngological patients.

	MLST	*spa* Type	*SCCmec* Cassette Type	ACME Type	Virulence and Biofilm Formation Genes
*S. aureus* WAW1257	ST 398 (*arcC* allel 3; *aroE* allel 35; *glpF* allel 19; *gmk* allel 2; *pta* allel 20; *tpi* allel 26; *yqiL* allel 39)	t4474	IVE	II	*clfB, clfA, fnbB, fib, hlg, hla, hld, hlb, icaABDC* operon
*S. haemolyticus* WAW954	ST42 (*arcC* allel 1; *cfxE* allel 1; *hemH* allel 1; *leuB* allel 1; *RiboseABC* allel 4; *SH1200* allel 1; *SH1431* allel 5)	not applicable	*mecA* only	III	*fib, hla, hlb*

**Table 2:** Antimicrobial susceptibility profiles and antibiotic resistance genes of *S. aureus* and *S. haemolyticus* strains.

	Strain No. WAW1257 (*S. aureus*)			Strain No. WAW954 (*S. haemolyticus)*		
Antibiotic	MIC (µg/mL)	Interpretation (S/R)	Antibiotic Restistance Genes	MIC (µg/mL)	Interpretation (S/R)	Antibiotic Restistance Genes
Linezolid	3	S	*optrA*, *poxtA*, T314C and G362A mutations in *rplC* (L3), C575T mutation in *rplD* (L4)	6	R	*cfr*
Cefoxitin	48	R	*mecA*	64	R	*mecA*
Vancomycin	3	R	-	4	S	-
Teicoplanin	3	R	-	6	R	-
Daptomycin	1	S	-	0.75	S	-
Fosfomycin	3	S	-	256	R	-
Ciprofloxacin	32	R	*norA*	32	R	-
Tetracycline	256	R	-	96	R	-
Tigecycline	0.50	S	-	1.5	R	-
Chloramphenicol	48	R	-	256	R	*fexA*
Gentamicin	0.75	S	-	24	R	*aac(6′)-Ie-aph(2″)*
Clindamycin	256	R	-	256	R	-
Amikacin	3	S	-	6	S	-
Erythromycin	256	R	-	256	R	-
Trimethoprim-Sulfamethoxazole	32	R	-	2	S	-
Levofloxacin	8	R	*norA*	32	R	-
Nitrofurantoin	96	R	-	128	R	-
Benzylpenicillin	24	R	-	256	R	-

MICs were determined by the E-test method. R: Resistance; S: Susceptibility.

**Table 3:** The nucleotide sequences of primers used for the detection of the virulence genes.

	Gene	Sequence (5′-3′)	Product Size (bp)	References
Adhesin genes	*clfB (S. aureus)*	ACATCAGTAATAGTAGGGGGCAAC	205	[88]
		TTCGCACTGTTTGTGTTTGCAC		
	*clfA (S. aureus)*	ATTGGCGTGGCTTCAGTGCT	292	
		CGTTTCTTCCGTAGTTGCATTTG		
	*fnbB (S. aureus)*	GTAACAGCTAATGGTCGAATTGATACT	524	
		CAAGTTCGATAGGAGTACTATGTTC		
	*fnbA (S. aureus)*	GTGAAGTTTTAGAAGGTGGAAAGATTAG	643	
		GCTCTTGTAAGACCATTTTTCTTCAC		
	*fib (S. aureus)*	CTACAACTACAATTGCCGTCAACAG	404	
		GCTCTTGTAAGACCATTTTCTTCAC		
	*fib (S. haemolyticus)*	TTATTTGACTTTCATACTTTGTA	1698	This study
		ATGGCATATGATGGCTTATTCA		
Hemolysin genes	*hla (S. aureus)*	CTGATTACTATCCAAGAAATTCGATTG	209	[89]
		CTTTCCAGCCTACTTTTTTATCAGT		
	*hlg (S. aureus)*	GTCAYAGAGTCCATAATGCATTTAA	535	
		CACCAAATGTATAGCCTAAAGTG		
	*hld (S. aureus)*	AAGAATTTTTATCTTAATTAAGGAAGGAGTG	111	
		TTAGTGAATTTGTTCACTGTGTCGA		
	*hlb (S. aureus)*	GCAATATAAACGCGCTGATTTAATCG	518	[90]
		GAGTGCCTTTATTGACATTAAGGTCG		
	*hla (S. haemolyticus)*	TGGGCCATAAACTTCAATCGC	72	[91]
		ACGCCACCTACATGCAGATTT		
	*hlb (S. haemolyticus)*	ATGTCTAACTCAACTAAGAATGC	684	This study
		CTAAATAAAATAAAGTATTGCTA		
*ica* operon (*S. aureus* and *S. haemolyticus*)	*icaA*	ACACTTGCTGGCGCAGTCAA	188	[92]
		TCTGGAACCAACATCCAACA		
	*icaB*	AGAATCGTGAAGTATAGAAAATT	900	[93]
		TCTAATCTTTTTCATGGAATCCGT		
	*icaC*	ATGGGACGGATTCCATGAAAAAGA	1100	
		TAATAAGCATTAATGTTCAATT		
	*icaD*	ATGGTCAAGCCCAGACAGAG	198	[92]
		AGTATTTTCAATGTTTAAAGCAA		

**Table 4:** Primer sequences and PCR conditions used to the amplification and sequencing of 23S rRNA, *rplC*, *rplD*, *rplV* genes.

Target Genes		Sequence (5′-3′)	PCR Conditions	Cycles (Steps 2–4)	Reference
*rplC* (L3) 822-bp	rplC-F (*S. aureus*)	AACCTGATTTAGTTCCGTCTA	94 °C for 2 min 94 °C for 1 min 50 °C for 1 min 72 °C for 1 min 72 °C for 5 min	33	[94]
	rplC-R	GTTGACGCTTTAATGGGCTTA			
	rplC-F (*S. haemolyticus*)	ACCCTGATTTAGTTCCGTCTA			[95]
*rplD* (L4) 1099-bp	rplD-F	TCGCTTACCTCCTTAATG	95 °C for 5 min 95 °C for 30 s 45 °C for 30 s 72 °C for 1 min 72 °C for 10 min	30	[94]
	rplD-R	GGTGGAAACACTGTAACTG			
*rplV* (L22) 520-bp	rplV-F	TTTCAGCATACCATTTTGCTTCC	94 °C for 2 min 94 °C for 10 s 50 °C for 30 s 72 °C for 30 s 72 °C for 5 min	30	[6]
	rplV-R	TAAAGGACATGCAGCAGACG			
23S rRNA 846-bp	23S-F	CGGCGGCCGTAACTATAACG	95 °C for 5 min 95 °C for 30 s 50 °C for 30 s 72 °C for 30 s 72 °C for 10 min	30	
	23S-R	CAGCACTTATCCCGTCCATAC			

## Data Availability

The datasets generated for this study can be found in Genbank, MW267294-MW267295; MW272559-MW272560; MW272562-MW272563; MW272556-MW272557.
